# Chloroplasts Protein Quality Control and Turnover: A Multitude of Mechanisms

**DOI:** 10.3390/ijms23147760

**Published:** 2022-07-14

**Authors:** Yunting Fu, Xifeng Li, Baofang Fan, Cheng Zhu, Zhixiang Chen

**Affiliations:** 1 College of Life Sciences, China Jiliang University, Hangzhou 310018, China; ting_2316156588@163.com (Y.F.); 19a0902115@cjlu.edu.cn (X.L.); 2 Department of Botany and Plant Pathology, Purdue University, West Lafayette, IN 47907-2054, USA; bfan@purdue.edu

**Keywords:** chloroplast protein quality control, chloroplast proteases, ubiquitin proteasome system, chloroplast associated protein degradation, chloroplast unfolded protein responses, autophagy, vesicle-mediated protein degradation

## Abstract

As the organelle of photosynthesis and other important metabolic pathways, chloroplasts contain up to 70% of leaf proteins with uniquely complex processes in synthesis, import, assembly, and turnover. Maintaining functional protein homeostasis in chloroplasts is vitally important for the fitness and survival of plants. Research over the past several decades has revealed a multitude of mechanisms that play important roles in chloroplast protein quality control and turnover under normal and stress conditions. These mechanisms include: (i) endosymbiotically-derived proteases and associated proteins that play a vital role in maintaining protein homeostasis inside the chloroplasts, (ii) the ubiquitin-dependent turnover of unimported chloroplast precursor proteins to prevent their accumulation in the cytosol, (iii) chloroplast-associated degradation of the chloroplast outer-membrane translocon proteins for the regulation of chloroplast protein import, (iv) chloroplast unfolded protein response triggered by accumulated unfolded and misfolded proteins inside the chloroplasts, and (v) vesicle-mediated degradation of chloroplast components in the vacuole. Here, we provide a comprehensive review of these diverse mechanisms of chloroplast protein quality control and turnover and discuss important questions that remain to be addressed in order to better understand and improve important chloroplast functions.

## 1. Introduction

Chloroplasts are organelles of plant and algal cells where light energy is converted into chemical energy through photosynthesis [1]. Chloroplasts also perform a variety of other metabolic processes in plants including the assimilation of nitrogen, phosphorus, and sulfur as well as the synthesis of diverse biologically important molecules in plant cells including fatty acids, membrane lipids, phenylpropanoids, isoprenoids, tetrapyrroles, starch, and hormones [2,3]. Chloroplasts contain a double-membrane envelope and a third membrane system, the thylakoids, which are in the chloroplast stroma and are organized as stacks of thylakoid discs called grana connected by intergranal thylakoids known as lamellae [4]. Chloroplasts also contain additional important structures such as starch grains, plastoglobules, and stromules [4]. As endosymbiotically-derived organelles from cyanobacteria, chloroplasts retain a minimal genome of about 100 genes encoding essential factors, including core genetic factors [5,6]. For the expression of the organellar genes, chloroplasts own eubacterial-type transcription and translation machineries [5,6]. The vast majority of the chloroplast proteins are encoded by nuclear genes and are transported post-translationally from the cytosol into the chloroplast through complex import machinery [7,8,9].

Maintaining functional protein homeostasis in chloroplasts is a constant challenge not only because of the vital roles but also because of the uniquely complex processes of synthesis, import, assembly, and turnover of chloroplast proteins in plant cells. First, chloroplasts contain up to 70% of the leaf proteins, a majority of which are photosynthetic proteins including ribulose bisphosphate, carboxylase/oxygenase (Rubisco), and chlorophyll binding/light harvesting complex proteins [10]. The failure or imbalance of quality control of the vast amount of chloroplast proteins is not only energetically costly but also potentially deadly and can directly affect plant fitness and survival [11,12]. Secondly, since a majority of chloroplast proteins are synthesized in the cytosol as precursors, the quality control of chloroplast proteins is highly complex, spanning multiple subcellular compartments from the cytosol to the chloroplast envelop, and to the intra-chloroplast sub-compartments [12,13]. Thirdly, chloroplasts are constantly exposed to a wide spectrum of environmental conditions. During photosynthesis, light energy constantly damages photosynthetic proteins, and the light-dependent generation of reactive oxygen species (ROS) leads to photooxidative stress, which also impairs protein functions [14,15]. Chloroplast protein structures and functions are also highly impacted by other abiotic and biotic stresses including extreme temperatures, drought, nutrient starvation, and pathogen infection [16,17]. As an important metabolic hub, chloroplast protein homeostasis directly affects chloroplast health critical for plant response to a variety of abiotic and biotic stresses [16,17]. Fourthly, since chloroplasts contain the majority of leaf proteins, their degradation during leaf senescence for the redistribution of released amino acids to other parts of the plant, including immature growing seeds, is a critical process for increasing the nitrogen use efficiency of plants, including crop plants [18,19]. Therefore, better knowledge of protein quality control and turnover in chloroplasts is critical for understanding how plants grow, develop, and respond to adverse environmental conditions, which is ultimately necessary to engineer crops with improved yield, quality, and stress tolerance.

Over the past several decades, great progress has been made in the understanding of dynamic protein quality control and turnover in chloroplasts under both normal and stress conditions. It has been well recognized that chloroplasts contain several types of intraplastidic proteolytic machineries derived from their endosymbiosed prokaryotic ancenstor that are important for chloroplast proteome biogenesis and homeostasis [11]. More recent research has revealed the critical role of the 26S ubiquitin–proteasome system (UPS) in the removal of unimported chloroplast precursor proteins in the cytosol, and chloroplast-associated protein degradation (CHLORAD) of the chloroplast protein translocation apparatus in response to developmental and environmental cues [12,20,21]. In addition, chloroplasts can unleash chloroplast-unfolded protein responses (cpUPR) upon increased accumulation of misfolded and aggregated proteins inside the organelles to increase protein-folding capacity and restore protein homeostasis [22]. Finally, vesicle-mediated delivery of chloroplast proteins or even entire chloroplasts to the vacuole for degradation occurs during sugar starvation, senescence, and oxidative stress [18,23]. In this review, we provide a comprehensive discussion of diverse pathways and mechanisms that participate in chloroplast protein quality control and turnover under both normal and stress conditions.

## 2. Intraplastidic Protease Complexes in Chloroplast Proteome Homeostasis

Three endosymbiotically-derived proteases, FtsH (filamentation temperature-sensitive H), Deg (degradation of periplasmic proteins) and Clp (caseinolytic protease), have vital roles in chloroplast protein homeostasis [11] (Table 1). FtsH is a metalloprotease that belongs to the AAA+ (ATPase associated with various cellular activities) protein super family [24]. In *E. coli*, FtsH is encoded by a single gene and forms a homo-hexamer. In plants, there are multiple FtsH isomers that form a hetero-complex and are associated with the thylakoid and envelope membranes. In Arabidopsis, there are 12 genes encoding FtsH isomers, nine of which are localized in the thylakoid membrane [11]. The FtsH hexamer consists of a chaperone ring and a proteolytic chamber that degrades the protein substrate and releases peptide products into the stroma. Four major Arabidopsis FtsH isomers (FtsH1, 2, 5 and 8) that form the FtsH hetero-complex can be functionally divided into two types: Type A (FtsH1 and 5) and Type B (FtsH2 and 8) [25,26,27] (Table 1). A deficiency of either FtsH2 or 5, or both, causes leaf variegation but the deficiency of both Type A (FtsH1 and 5) or Type B (FtsH2 and 8) leads to seedling lethal phenotype [27]. In most land plants and alga, the two-type FtsH isomers are well-conserved. 

Thylakoid FtsH is involved in photosystem II (PSII) repair [28,29] (Table 1). The D1 protein, one of the two proteins of the PSII reaction center, is a major target of photodamage and is under rapid turnover, which prevents the entire PSII from being photodamaged. The rapid turnover of the D1 protein known as the PSII repair cycle is achieved by the specific and rapid degradation of D1 and the replacement of newly synthesized D1 protein molecules [28,29]. Both in vitro experiments and in vivo genetic evidence using FtsH2 or FtsH5-deficient mutants indicate that FtsH plays a predominant role in light-dependent D1 degradation and PSII repair [30,31,32]. As will be discussed later, chloroplast DEG protease also plays a role D1 degradation. In addition to PSII repair, chloroplast FtsH proteases participate in other important processes in chloroplasts, including unassembled protein degradation, thylakoid formation, and heat-stress responses [27,32,33,34]. 

Clp is an ATP-dependent serine protease complex mostly localized in the stroma with small fractions at the inner envelope [11]. Like FtsH, the Clp complex has two functional units: a hexameric ring-like ATP-dependent chaperone unit and a barrel-shaped tetradecadmeric proteolytic core with serine-histifine-aspartate catalytic triads (Table 1). The chaperone unit subunits ClpC1, C2, and D form individual homohexamers that recognize specific protein substrates with or without the aid of the adaptor protein ClpS and the interacting protein ClpF, and unfold and translocate them into the proteolytic core for degradation (Table 1). The tetradecameric proteolytic core consists of two rings: the P ring containing Clp3, 4, 5, and 6 subunits, and the R ring containing ClpP1, R1, R2, R3, and R4 subunits (Table 1). Unlike ClpP subunits, ClpR subunits lack the catalytic serine-histidine-aspartate catalytic triads. The tetradecameric proteolytic core also contains plant-specific ClpT1 and T2 accessory proteins for core complex assembly and stabilization [35,36] (Table 1). Clp targets multiple chloroplast proteins including a copper-transporting P-type ATPase, an isoprenoid pathway enzyme, and tetrapyrrole metabolic enzymes [37,38,39,40,41]. The Clp protease also interacts with an inner chloroplast envelop protein Tic110, possibly to regulate the quality control of protein translocation across the inner membrane [42,43,44]. The critical role of Clp in chloroplast protein homeostasis is also demonstrated by the activation of cpUPR upon the inhibition or reduction of Clp activity, which will be discussed below in more detail.

Deg is an ATP-independent serine protease with a protease domain containing a catalytic histidine-aspartate-serine triad and PDZ (post-synaptic density protein, Drosophila disc large tumor suppressor 1, and zonula occludens-1 protein) domains required for protein–protein interactions and recognition of protein substrates [45]. There are 16 genes encoding Deg proteins in Arabidopsis and at least five of them are localized in chloroplasts: Deg 1, 5, and 8 in the thylakoid lumen, Deg2 on the stromal side of the thylakoid membrane, and Deg7 in the stroma [46,47,48,49] (Table 1). Deg proteins from *E. coli* and human form homo-trimers and their further association leads to the formation of hexamers [50]. Arabidopsis Deg1 and Deg2 also form homotrimers that subsequently assemble into a homo-hexamer through trimer dimerization. Significantly, the dynamic structural conversions of Deg1 from its inactive monomers to the active hexamers is stimulated upon lumenal acidification under photoinhibitory light conditions [46]. The dimerization of Deg2 trimers into hexamers, on the other hand, is stimulated by the binding of peptide ligands [51]. Deg5 and Deg8 can assemble into heterohexamers and act synergistically [52,53]. The luminal Deg1, 5, and 8 are involved in the degradation of D1 protein and PSII repair. The suppression of *Deg1* expression led to increased light sensitivity of PSII activity and reduced D1 degradation [49,54]. Likewise, Deg5- and 8-deficient mutants were viable but impaired in D1 degradation under strong light [53]. By contrast, a mutant deficient in the stromal Deg2 is normal in PSII photoinhibition, arguing against its role in D1 degradation and PSII repair [55].

## 3. CHIP-Mediated Cytosolic Degradation of Chloroplast-Targeted Precursors

CHIP (carboxyl-terminal Hsp70-interacting protein) is a highly conserved E3 ligase that is bound directly to Hsp70, Hsp90, and ubiquitinate Hsp70/90-bound proteins for degradation by the UPS [56]. In metazoans, CHIP has been extensively investigated for its critical role in protein quality control. Like their counterparts in metazoans, plant CHIP contains an N-terminal tetratricopeptide repeat (TRP) and a C-terminal U-box domain [57,58]. The CHIP TRP domain binds directly to the conserved EEVD motifs at the C-terminus of heat shock cognates (Hsc)/Hsp70 and Hsp90 [59,60]. The C-terminal U-box domain of CHIP possesses both E3 ubiquitin ligase and polyubiquitin-chain extension activities [61]. Hsp70 and Hsp90 act as molecular chaperones that normally promote protein folding and maturation [62]. However, they also recognize different types of misfolded proteins and promote their degradation through coordinated action with chaperone-dependent ubiquitin E3 ligases such as CHIP. The switch in the role of Hsp70 and Hsp90 is apparently achieved through the action of CHIP as a co-chaperone by decreasing the ATPase activity of both Hsp70 and Hsp90 and inhibiting the Hsp70/90-mediated protein folding cycle [56,63]. 

**Figure 1:**
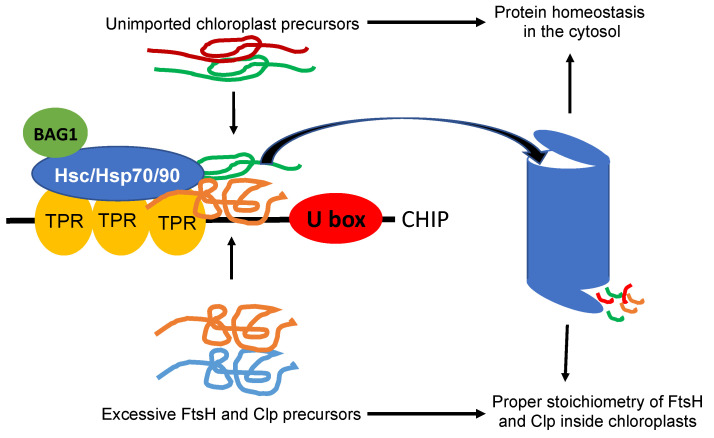
CHIP-mediated chloroplast protein quality control. Molecular chaperones Hsc/Hsp70/90 recognize unimported chloroplast precursors and can switch their role to facilitate protein degradation upon binding the PTR domain of CHIP. CHIP can also directly recognize some chloroplast precursors such as excessive FtsH and Clp precursors. CHIP mediates the ubiquitination in these unwanted or excessive chloroplast precursors and targets their degradation by the 26S proteasome. CHIP-mediated degradation of unimported chloroplast precursor proteins prevents the accumulation of misfolded proteins and helps maintain protein homeostasis in the cytosol. The degradation of excessive FtsH and Clp protein subunits may help maintain proper stoichiometry of these protease complexes inside chloroplasts.

CHIP plays a direct role in chloroplast protein quality control by targeting the degradation of chloroplast-destined precursors to prevent their accumulation in the cytosol (Figure 1). In Arabidopsis mutant plants defective in protein import into chloroplasts, the genes encoding cytosolic Hsc70-4, CHIP, and BAG1 (B-cell lymphoma-2 associated athanogene 1) co-chaperone are strongly induced [64]. Hsc70-4 recognizes specific sequences in the transit peptides of unimported protein precursors. CHIP interacts with Hsc70-4 and functions as an E3 ligase in Hsc70-4-mediated protein degradation by the UPS. The suppression of Hsc70-4 or the expression of a dominant negative CHIP mutant protein reduces degradation of chloroplast precursor proteins in the cytosol [64]. In mammalian cells, BAG proteins function as nucleotide-exchange factors for Hsc70 and play important roles in protein homeostasis [65]. Arabidopsis BAG1 also plays a crucial role in Hsc70-4/CHIP-mediated proteasomal clearance of misfolded and unimported plastid proteins in the cytosol [66].

CHIP also plays an indirect role in chloroplast protein quality control through the regulation of chloroplast proteases (Figure 1). CHIP interacts with the proteolytic subunits Clp3, 4, and 5 of the chloroplast Clp protease in yeast and ubiquitinates them in vitro [67,68]. CHIP also interacts with FtsH1 and FtsH2 in vivo and ubiquitinates them in vitro [69]. The overexpression of CHIP reduces the steady-state levels of these chloroplast protease subunits and the degradation of the D1 PSII reaction center protein [69]. These chloroplast protease subunits are all encoded by the nuclear genes and are synthesized in the cytosol as precursors before being imported into the chloroplasts for folding and assembly. In CHIP-overexpression lines, CHIP acts in coordination with Hsp70 molecular chaperones for the increased degradation of Clp and FtsH subunits in the cytosol. The targeting of chloroplast proteases important for chloroplast protein quality control by CHIP is counterintuitive given the cytoprotective role of CHIP. A plausible explanation for the seemingly contradictory findings about the phenotypes of the CHIP-overexpressing plants could be the unphysiologically high levels of CHIP proteins in these transgenic lines that lead to the non-specific degradation of native proteins. As discussed earlier, both ClpP and FtsH proteases are heteromeric AAA ATPase complexes composed of related ClpP and FtsH subunits, respectively. The suppression or overexpression of ClpP4, one of the ClpP core subunits, led to chlorotic phenotypes in Arabidopsis, indicating that proper stoichiometry of different ClpP and FtsH subunits in their respective protease complexes could be important for their stability or activity [68]. Interestingly, the overexpression of CHIP could rescue the chlorotic phenotypes of both ClpP4 antisense and overexpressing plants [68]. The unbalanced levels of Clp core proteins in ClpP4 antisense and overexpressing plants were restored to wild-type levels by CHIP overexpression [68]. These results suggest that CHIP may play a critical role in maintaining the proper stoichiometry of different ClpP and FtsH subunits in their respective protease complexes through the selective degradation of ClpP and FtsH subunits, thereby positively regulating the homeostasis of ClpP and FtsH proteolytic subunits to protect chloroplast functions. In transgenic tobacco plants, a similar role of CHIP in the regulation of the chloroplast proteases has also been demonstrated [68].

The critical role of CHIP in chloroplast protein quality control is also apparent under heat stress conditions. CHIP genes from both Arabidopsis and tomato are induced by heat treatment, and mutations of the Arabidopsis CHIP gene, or the virus-induced silencing of the tomato CHIP gene, leads to increased heat sensitivity which is associated not only with the increased development of heat stress symptoms but also with the rapid reduction of photosynthesis and increased ion leakage under high temperatures [21,70]. It is known that the capacity of protein precursor translocation into chloroplasts decreases at high temperatures, resulting in their increased accumulation in the cytosol [71]. High temperatures could also increase the misfolding of chloroplast precursor proteins. CHIP could play a critical role in heat tolerance by targeting the degradation of these unfolded or misfolded chloroplast precursor proteins in the cytosol. This possibility is consistent with the finding that there is increased accumulation of insoluble chloroplast protein aggregates in the *chip* mutant when compared with that in wild-type plants under high temperatures [70]. Interestingly, CHIP can interact with two of the most abundant nuclear-encoded proteins, Rubisco small (RbcS) and Lhcb6 [72], further underscoring the role of the E3 ligase in chloroplast protein quality control. Furthermore, heat stress could lead to the increased misfolding of ClpP and FtsH protease subunit precursors and the disruption of the proper stoichiometry of the protease precursors due to differential stability under heat stress. CHIP could positively regulate the homeostasis of the proteolytic subunits to promote the activity and stability of the protease complexes under heat stress. These chloroplast proteases also degrade heat-denatured proteins in chloroplasts, and consequently their loss-of-function mutants are not only impaired in photosynthetic capacity, but are also highly susceptible to elevated temperatures [33].

## 4. Chloroplast-Associated Protein Degradation (CHLORAD)

The vast majority of chloroplast proteins are encoded by nuclear genes [20]. These chloroplast-destined proteins are synthesized in the cytosol as precursors with an N-terminal transit peptide and imported into chloroplasts through translocon complexes in the outer (TOC) and inner (TIC) envelope membrane. The levels of chloroplast protein import change greatly in response to developmental stages, environmental stimuli, and stress conditions in order to provide an optimal chloroplast proteome and organellar functions [73,74,75]. Recent research has revealed that ubiquitin-dependent turnover of chloroplast TOC proteins plays a critical role in the regulation of chloroplast protein import [12,20]. 

The core TOC complex contains three proteins: Toc159, Toc34 (Toc33 in Arabidopsis), and Toc75 (the numeric parts of the protein names are based on their size in kilodaltons) [20]. TOC159 and TOC33 are receptors with cytosolic GTPase domains that recognize the signal peptides of precursor proteins. TOC159 contains an intrinsically disordered N-terminal acid domain, a central GTPase domain, and a large C-terminal membrane-anchoring domain. Toc33 contains an N-terminal GTPase domain and a C-terminal membrane-spanning domain. In higher plants, both Toc159 and Toc34 are encoded by gene families and are present in multiple isoforms. In Arabidopsis, Toc159 has three homologs: Toc132, Toc120, and Toc90, while Toc33 has a single homolog, Toc34 [76]. Genetic evidence indicates that Toc159 and Toc33 form a group of receptors responsible for the import of photosynthetic preproteins and/or preproteins belonging to age-dependent group I, whereas Toc132/120 and Toc34 are responsible for the import of non-photosynthetic, housekeeping proproteins, and/or those belonging to age-dependent group II [77,78,79,80,81,82,83]. Like Toc75, SP2 belongs to the Omp85 superfamily of proteins but without the POTRA domain. SP2 can form a channel to assist TOC protein translocation across the chloroplast outer membrane [20,76]. Unlike the Toc159 and Toc33, Toc75 is encoded by a single gene in Arabidopsis. 

**Figure 2:**
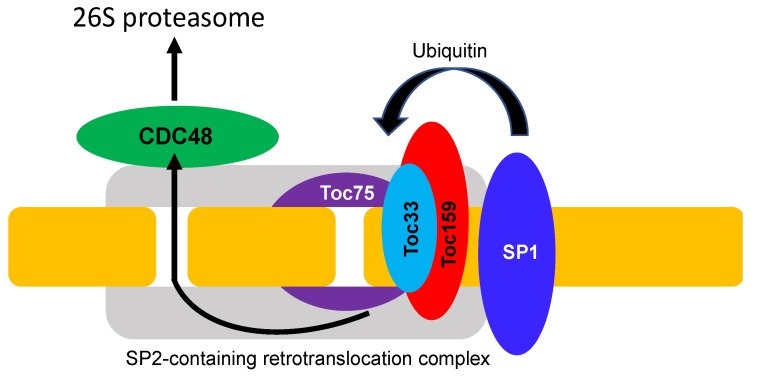
CHLORAD of TOC complex components. The RING-type E3 ubiquitin ligase SP1 directs the ubiquitination of TOC complex subunits. SP2-containing retrotranslocation and CDC48 complexes act to extract ubiquitinated TOC complex subunit proteins to the 26S proteasome in the cytosol for degradation.

As expected, mutants for Toc33 and Toc159 in Arabidopsis (also known as *plastid protein import* or *ppi1* and *ppi2* mutants, respectively) display strong defects in chloroplasts with albino or chlorotic leaf phenotypes [77,80,81,83]. Forward genetic analysis has identified a second-site suppressor of *ppi1* (*sp1*) that can partially suppress the chlorotic phenotypes, and improves the chloroplast ultrastructural organization and the protein import capacity of *ppi1* [84]. SP1 is a RING-type E3 ubiquitin ligase with two transmembrane domains. SP1 interacts with all TOC proteins and targets their degradation by ubiquitination (Figure 2). In the *ppi1* mutant background deficient in Toc33, the mutation of SP1 increases the abundance of other TOC proteins to enhance the greening and growth of the *ppi1* mutant [84]. Suppressor screens also identified another protein, SP2, in ubiquitination-mediated degradation of TOC proteins [85]. Like Toc75, SP2 belongs to the Omp85 superfamily of proteins but with the POTRA domain. SP2 can form a channel to assist TOC protein translocation across the chloroplast outer membrane before degradation by the UPS in the cytosol [85] (Figure 2). The energy for extracting TOC proteins through the SP2 channel in the chloroplast outer membrane is provided by the cytosolic CDC48 (cell division cycle 48) ATPase (Figure 2), which also functions as a core motor component in the extraction of misfolded endoplasmic reticulum (ER) proteins across the ER membrane in ER-associated protein degradation (ERAD) [86]. The process of ubiquitination-dependent TOC protein degradation through coordinated action of the SP1, SP2, and CDC48 has been referred to as chloroplast-associated protein degradation or CHLORAD [20,85] (Figure 2). 

There is direct genetic evidence for the important role of CHLORAD in the reconfiguration of chloroplast proteome in development and in response to environmental cues and stress conditions. For example, upon exposure to light, heterotrophic etioplasts in etiolated seedlings rapidly differentiate into chloroplasts after seed germination in the soil. The *sp1* mutants display inefficient etiolation, associated with imbalance in TOC receptor levels, delayed chloroplast differentiation, and reduced accumulation of photosynthetic proteins [85]. The *sp1* mutants are also defective in the speed of senescence, associated with attenuated transformation of chloroplasts to gerontoplasts during the transition [85]. Similarly, tomato SP1 homologs regulate both dark-induced and age-related leaf senescence [87]. In addition, CHLORAD plays an important role in tomato fruit ripening, in which chloroplasts are converted into chromoplasts for the accumulation of carotenoid pigments [87]. The suppression of expression for tomato SP1, or its homolog SPL2, delays tomato fruit ripening, while SP1 overexpression promotes ripening [87]. Arabidopsis SP1 also plays a critical role in plant responses to abiotic stress. Arabidopsis *sp1* mutants are hypersensitive to salt (NaCl), osmotic (mannitol), and oxidative (paraquat) stresses, whereas SP1-overexpressing plants have increased tolerance [88]. SP1 promotes abiotic stress tolerance most likely by reducing TOC proteins under stress conditions to limit the import of photosynthetic proteins, thereby attenuating photosynthetic activity and reducing potential ROS production and photo-oxidative damage [88].

TOC proteins are also subjected to the regulation of other proteolytic pathways in addition to SP1-dependent CHLORAD. During seed germination, proplastids transform into chloroplasts, and this transition is associated with increased levels of plant hormone gibberellic acid (GA) [89]. Under unfavorable germination conditions with low levels of GA, the DELLA protein, a negative regulator of GA signaling, interacts with Toc159 and promotes Toc159 ubiquitination and degradation by the UPS in an SP1-indenedent manner to delay the onset of chloroplast biogenesis at the early germination stage [90]. A more recent study has further revealed that the turnover of Toc159 by the UPS cross-talks with the small ubiquitin-related modifier (SUMO) pathway. TOC159 interacts with the E2 SUMO conjugating enzyme (SCE2) in yeast cells and also contains an E3 SUMO ligase (SUMO3)-interacting motif (SIM) in the GTPase domain and a SUMO3 covalent SUMOylation site in the membrane domain [91]. Subsequent analysis has shown that Toc159 SUMOylation protects Toc159 from UPS-dependent degradation prior to TOC assembly [91]. On the other hand, like the sp1 mutants, Arabidopsis mutants for a number of components in the SUMO pathway including SUMO2 and SUMO3 can partially suppress the phenotypes of the *ppi1* mutant for Toc33 through improving chloroplast development and enhancing accumulation of key Toc proteins [92]. Therefore, the SUMO system can both stabilize and destabilize key Toc proteins, probably dependent on different biological contexts. It is also possible that the SUMO system can have differential effects on the stability of Toc precursor proteins in the cytosol versus those membrane-bound Toc proteins.

There is genetic evidence that the degradation of chloroplast precursors by the cytosolic UPS occurs even in wild-type plants with no defect in chloroplast import. Mild impairment of the proteasome leads to increased abundance of precursor proteins in the cytosol, elevated accumulation of functional photosynthetic complexes in chloroplasts, and improved photosynthetic performance [93]. These results indicate that even functional chloroplast precursors are subjected to continuous degradation by CHLORAD in the cytosol, which constrains chloroplast biogenesis and function, including photosynthesis [93]. These new insights into the regulatory network of chloroplast precursor quality control, import, and assembly present potential strategies to improve photosynthesis by stabilizing chloroplast precursor proteins through various means. 

Sex-linked CHLORAD is involved in the uniparental transmission of chloroplast DNA [94]. In the green algae *Chlamydomonas reinhardtii*, mating combines isomorphic plus and minus gametes, but chloroplast DNA from plus gametes is selectively maintained in zygotes. OTU2p (otubain protein 2), an otubain-like deubiquitinase encoded in the plus mating type locus MT+, protects the plus chloroplast from the degradation of the TOC preproteins by the UPS during gametogenesis [94]. The knockout of OTU2p and proteasome inhibitor treatment decreases TOC levels and redirects selective DNA degradation in chloroplasts in a mating type-independent manner, confirming that plus-specific Otu2p determines uniparental chloroplast DNA inheritance [94].

## 5. Chloroplast Unfolded Protein Response (cpUPR)

**Figure 3:**
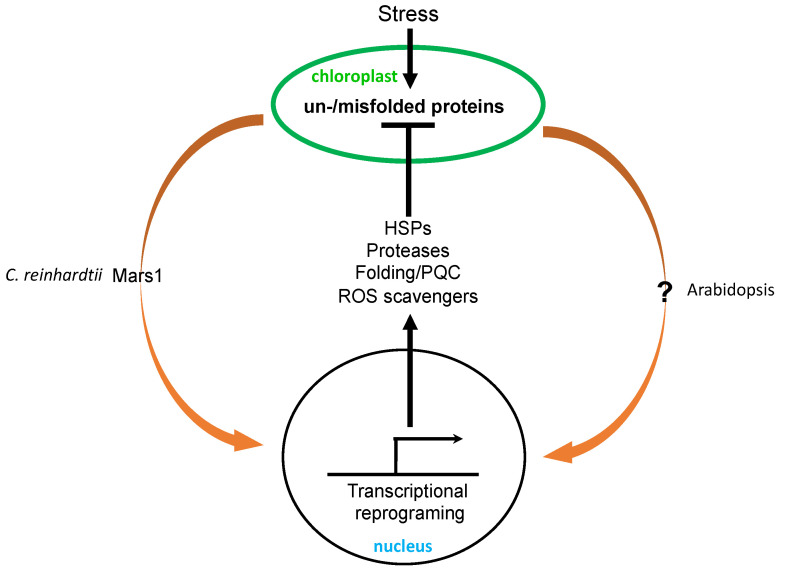
Chloroplast unfolded protein response (cpUPR). Stress such as high light intensity and deficiency of plastidic proteases leads to increased accumulation of unfolded and misfolded proteins in chloroplasts, which trigger cpUPR associated with activated transcription of genes encoding HSPs, proteases, and proteins involved in protein folding and quality control and ROS scavenging. Increased production of these proteins promotes protein folding, and the removal of misfolded proteins and ROS to protect chloroplast functions. In green algae *C. reinhardtii*, the Mars1 kinase in the cytosol is required for cpUPR. In Arabidopsis, the signaling mechanisms and components involved in the chloroplast-to-nucleus communication for activation of cpUPR have not been firmly established.

The unfolded protein response (UPR) is a cellular stress response to increased accumulation of unfolded and misfolded proteins to restore protein homeostasis [95]. It has been best analyzed in the ER and, to a lesser extent, in mitochondria. In plants the UPR elicited by ER stress is mediated by two signaling pathways: one involving inositol-requiring enzyme 1-basic leucine zipper 60 (IRE1-bZIP60) and the other involving the membrane transcription factors bZIP17 and bZIP28 [96,97]. Cellular UPR activation is associated with increased expression of nuclear genes encoding protein chaperones, proteases, and other proteins involved in folding, trafficking, and quality control [95]. Chloroplasts synthesize some of their own proteins and import most of their proteins from the cytoplasm to perform their roles. As in the ER and mitochondria, the newly synthesized or imported chloroplast proteins must be rapidly processed inside the chloroplasts. Under certain stress conditions such as high temperature and high light intensity, protein folding and quality control inside chloroplasts can become compromised, leading to increased accumulation of unfolded and misfolded proteins, which are highly toxic and must be detected and removed to prevent the organelle from proteotoxicity (Figure 3). 

Chloroplast UPR (cpUPR) has been analyzed only relatively recently in part because of the lack of effective methods for the specific and gradual induction of accumulation of unfolded and misfolded proteins inside chloroplasts. Such a method has been developed in the green algae *C. reinhardtii* through the conditional repression of the chloroplast *ClpP1* gene, leading to the gradual decrease of the ClpP1 subunit of chloroplast Clp protease complexes important for the degradation of damaged proteins [98]. Using this repressible system, Ranmindo and colleagues have demonstrated that a selective gradual reduction of ClpP leads to the alteration of a range of cellular structures including chloroplast morphology and the formation of vesicles characteristic of autophagosomes [98]. Moreover, transcriptome and proteome analysis during ClpP depletion revealed increased accumulation not only at the protein level but also at the RNA level, of small heat shock proteins, chaperones, proteases, and proteins involved in thylakoid maintenance [98,99] (Figure 3). Thus, the activation of a nuclear transcription program upon the perturbation of plastid protein homeostasis indicates a chloroplast-to-nucleus signaling pathway involved in organelle quality control, representing a cpUPR similar to the UPR in the ER and mitochondria [98,99].

The first component involved in cpUPR signaling in the algae *C. reinhardtii* was reported less than three years ago [100]. The signaling component Mars1 (mutant affecting in chloroplast-to-nucleus retrograde signaling 1) was identified through a genetic screen using a florescent reporter of a cpUPR-induced gene. Mars1 is a cytoplasmic protein kinase with a critical role in the activation of cpUPR, including the induction of cp-UPR-responsive genes, and its kinase activity is required for this role [100] (Figure 3). The mutant is highly susceptible to high light levels and ROS, both of which damage proteins inside the chloroplasts and can trigger cpUPR. By contrast, mutant algae expressing a dominant active version of the Mars1 kinase protein displayed increased tolerance to both high light levels and ROS [100]. These findings not only support the critical role of chloroplast-to-nucleus retrograde signaling in the activation of cpUPR for the protection of chloroplast functions, but also open a potential avenue to improve photosynthetic efficiency under stress conditions through the exploitation of cpUPR [22].

In Arabidopsis, the reduced accumulation of chloroplast proteases also causes chloroplast stress, which can in turn trigger cpUPR. Arabidopsis T-DNA insertion mutants for ClpC1, a molecular chaperone of the Hsp100 family and a subunit of Clp protease display phenotypes of chlorotic leaves, impaired photosynthesis and retarded growth [101]. Despite the proposed role of ClpC1 in chloroplast protein import and the degradation of misfolded precursors, these activities appeared to be largely normal in the *clpC1* mutants, probably due to the increased accumulation of ClpP paralogs and other stromal chaperones such as Cpn60, Hsp70, and Hsp90 [101]. Likewise, a T-DNA insertion mutant for ClpR2 displays a pale-green phenotype, smaller chloroplasts, decreased thylakoid accumulation, and retarded shoot development [102]. Large scale comparative proteomic analysis revealed the upregulation of chloroplast unfoldase ClpB3, other chaperones, proteases, and protein sorting components in the *clpR2* mutant [103] (Figure 3). Similarly, in Arabidopsis the *ftsH2* mutants accumulate increased levels of damaged chloroplast proteins in chloroplasts, which also trigger increased accumulation of heat-shock proteins, chaperones, proteases, and ROS detoxifiers [104] (Figure 3).

Relatively little is known about the signaling pathways that mediate cpUPR in higher plants (Figure 3). In Arabidopsis, treatment with plastid protein synthesis inhibitor lincomycin (LIN) can increase the accumulation of aggregated proteins in the chloroplasts, including the misfolding- and aggregation-prone deoxyxylulose 5-phosphate synthase (DXS), which catalyzes the first and main rate-limiting step of the methylerythritol 4-phosphate (MEP) pathway required for the production of plastidial isoprenoids [105]. Misfolded DXS proteins are substrates of Clp proteases, and their accumulation upon LIN treatment is likely due to the inhibited synthesis of the plastome-encoded ClpP1 subunit [105]. LIN-induced protein aggregation in chloroplasts also unleashes a specific retrograde signaling pathway that up-regulates the expression of ClpB3 and other nuclear genes encoding plastidic chaperones [105]. LIN-induced cpUPR is associated with increased expression of the heat shock transcription factor HsfA2. LIN-induced expression of HsfA2 and cpUPR-related target genes is normal in the mutant of GUN1 (genome uncoupled 1), a chloroplast-localized pentatricopeptide repeat (PPR) protein with a C-terminal small mutS-related (SMR) domain that plays a central role in the retrograde communication of chloroplasts with the nucleus. However, mutants defective in both GUN1 and plastid gene expression or protein quality control are lethal [105], indicating that the GUN1 is important for chloroplast protein homeostasis. This role of GUN1 is consistent with its interaction not only with proteins involved in plastidial gene expression transcription but also with proteins such as Hsp70 and ClpC chaperones with roles in chloroplast protein quality control. Likewise, HsfA2 is up-regulated in the mutant for FtsH2 [104].

## 6. Vesicle-Mediated Vacuolar Degradation of Chloroplast Proteins

**Figure 4:**
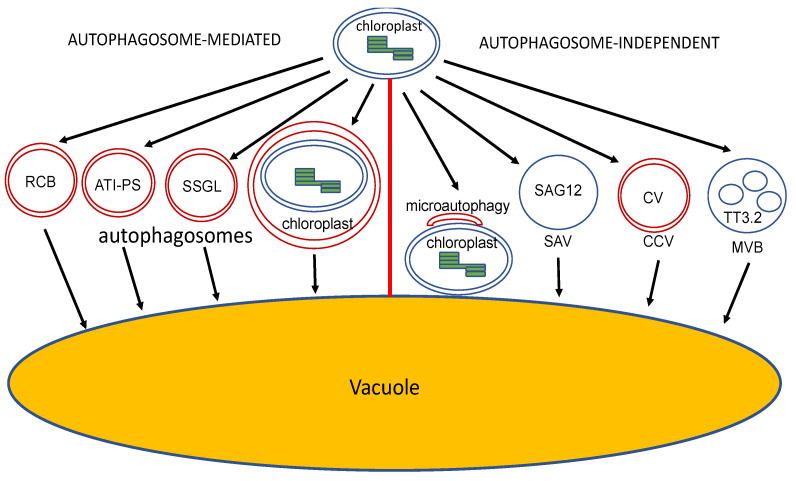
Diverse pathways for vesicle-mediated vacuolar degradation of chloroplasts and chloroplast components. Some of the pathways for degradation of chloroplast components are mediated by autophagosome-derived vesicles such as RCBs, ATI –PS and SSGL bodies. Whole chloroplasts can also be delivered by autophagosomes to the vacuole for degradation. Damaged chloroplasts can also be degraded in the vacuole by microautophagy in which the chloroplasts are isolated and delivered to the vacuole through direct enclosing. Chloroplast components can be delivered by vesicles other than autophagosomes to the vacuole for degradation. These vesicles include SAVs, CCVs and MVBs.

In addition to the plastidial proteases and extraplastidial UPS, chloroplast proteins can also be delivered by membrane-bound vesicles to the vacuole for degradation during senescence, and responses to nutrient (N and C) starvation and other stresses (Figure 4). Some of these pathways for the vacuolar degradation of chloroplast proteins are mediated by autophagosomes for cargo delivery, whereas others are independent of autophagosomes (Figure 4).

### 6.1. Autophagosome-Mediated Vacuolar Degradation of Chloroplast Components 

Autophagy is a highly conserved cellular process for the degradation of unwanted or damaged cytoplamsic components including organelles, proteins, and RNAs under both normal and stress conditions in eukaryotic cells [106,107]. Under normal growth conditions, autophagy occurs at low basal levels. Under stress conditions such as starvation, autophagy is induced with the formation of an isolation membrane known as phagophore, which can expand to sequester cytoplasmic cargos within a double-membrane vesicle called autophosome [108]. Upon fusion with the lysosomes/vacuoles, the mature autophosome can deliver the cargo to the lytic compartment for degradation. The core process of autophagosome formation requires more than 40 largely conserved autophagy-related (ATG) proteins. While autophagy may involve non-selective degradation of intracellular contents under certain conditions such as nutrient starvation, the broad roles of autophagy are mediated through selective removal of specific cellular components [109]. Selective autophagy relies on ubiquitin-like ATG8. Upon attachment with a phosphatidylethanolamine (PE) at its C-terminus, ATG8 is anchored in the membrane of autophagosomes and can act as a docking platform for cargo capture through interaction with selective autophagy receptors. Most selective receptors interact with autophagosome-anchored ATG8 through ATG8-interacting motifs (AIMs) with a W/Y/F-X-X-L/I/V sequence [110]. Other selective autophagy receptors interact with ATG8 through a ubiquitin-interacting motif (UIM)-like sequence [111].

Chloroplasts, which are abundant in proteins and other molecules, are dismantled during leaf senescence and under N or C starvation, and their constituents are transported to vacuoles for degradation by several autophagosome-related vesicles, including the Rubisco-containing bodies (RCBs) [112], the selective autophagy receptor ATI1 (ATG8-interacting Protein 1)-plastid associated (ATI-PS) bodies [113], and small starch granule-like (SSGL) bodies [114,115] (Figure 4). In addition, when cells are subjected to UV-induced damage, entire chloroplasts can be engulfed by autophagosomal structures [116,117] (Figure 4).

#### 6.1.1. RCBs

Autophagosome-like RCBs transport chloroplast stromal proteins, including Rubisco, to the vacuole for degradation [112] (Figure 4). The colocalization of RCBs labeled by a chloroplast-targeted red fluorescent protein with the GFP-ATG8 autophagosome marker supports the autophagic nature of RCBs. Furthermore, in both Arabidopsis and rice, the formation of RCBs is dependent on ATG genes including ATG5 and ATG7 [112,118]. These findings establish the autophagic nature of RCBs, which is also consistent with the high inducibility of RCBs by dark-induced carbon starvation and senescence [119,120]. 

Interestingly, certain components involved in the biogenesis of multivesicular bodies (MVBs) play a direct role in the degradation of chloroplast RCB cargo in the vacuole [121]. MVBs are specialized endosomes in the endocytic pathway that function in the internalization, transport, sorting, and degradation of plasma membrane proteins [122]. MVBs contain intraluminal vesicles generated from invagination and budding of the limiting membrane through the action of protein complexes named ESCRT-0, I, II, and III (endosomal sorting complex required for transport) [122]. The ESCRT-III subunit paralogs CHMP1A (charged MVB protein1) and CHMP1B are required for the transport of RCB cargo into the vacuoles [121]. Autophagy was induced, but autophagic degradation of the stromal cargo of RCBs was greatly reduced in the *chmp1* plants upon starvation. Thus, the autophagy process for releasing RCB bodies containing stromal proteins into the cytoplasm is normal, but the subsequent delivery of RCB bodies to the vacuole is defective in the chmp1 mutants [121]. Thus, two related partners in the lysosomal/vacuolar protein degradation system coordinate in the delivery of chloroplast proteins to the vacuoles for degradation during starvation.

#### 6.1.2. ATI-PS Bodies

Arabidopsis ATG8-interacting ATI1 and ATI2 are two closely related proteins that are unique to plants [123]. ATI1 and ATI2 are transmembrane proteins with long N-terminal intrinsically disordered regions (IDRs), which contain a functional AIM that binds ATG8. Under normal growth conditions, ATI1 and ATI2 are partially associated with the ER membrane network. Under carbon starvation, ATI1 and ATI2 become primarily associated with spherical compartments that dynamically move along the ER network and traffic to the central vacuole [113]. Following carbon starvation, ATI1 is also located on ATI1-PS bodies in the periphery and inside of plastids in senescing cells undergoing plastid degradation (Figure 4). ATI1-PS bodies contain thylakoid membrane proteins, chlorophylls, and other plastid components and are released from chloroplasts into the cytosol independent of the autophagic machinery. However, ATI1 on the plastid bodies interacts with ATG8f, and their fusion with the central vacuole is dependent on functional autophagy [113]. How ATI1 is re-localized from the ER under normal growth condition into chloroplasts under starvation is currently unknown.

#### 6.1.3. SSGL Bodies

Starch is a major product of photosynthesis in chloroplasts with an important role in carbohydrate metabolism in leaves through its biosynthesis and degradation. Starch is produced and stored in starch granules in plastids. Under carbon starvation in the dark, however, granule-like structures are also present in the cytosol and sequestered into the autophagic bodies [114,115] (Figure 4). These SSGL bodies contain granule-bound starch synthase I, a starch granule marker, and also colocalized with ATG8f. As with RCBs and ATI-PS bodies, the number of vacuole-localized SSGL bodies is drastically reduced when ATG6 is silenced [114,115]. Autophagy deficiency also leads to excessive starch accumulation in plant leaves [114]. These results indicate that autophagy plays a critical role in daily starch degradation by sequestering SSGL bodies to the vacuole for their metabolism. 

#### 6.1.4. Autophagy Degradation of Whole Chloroplasts

Under dark-induced senescence or upon exposure to UV-B light, whole chloroplasts are delivered to the vacuoles for degradation by autophagosomal structures [117] (Figure 4). These ATG8-labeled structures are much larger than typical autophagomes, allowing them to capture the whole chloroplasts and sequester them into a mature autophagsomes (Figure 4). They can also be readily detected in the vacuole, indicating their role for the autophagic degradation of entire chloroplasts. Furthermore, delivery of entire chloroplasts to the vacuole is reduced in the autophagy mutants, which is associated with increased UV sensitivity [117]. Exposure to UV-B light leads to ROS accumulation and chloroplast damage. Autophagy functions to remove UV-damaged chloroplasts as a protective mechanism to promote survival of plant cells. In Arabidopsis mutants for plastid ferrocheltase 2 (FC2), which is required for chlorophyll synthesis, there is also increased accumulation of ROS and degradation of damaged chloroplasts [124]. The PUB4 E3 ubiquitin ligase is required for the ubiquitination of the chloroplast surface and degradation of damaged chloroplasts [124]. It is unknown whether PUB4-dependent degradation of damaged chloroplasts also involves autophagy.

### 6.2. Autophagosome-Independent Vacuolar Degradation of Chloroplast Components

While autophagosome-dependent degradation of cytoplasmic constituents (also called macroautophagy) has been well established in chloroplast protein quality control and turnover, there are several autophagosome-independent pathways that also mediate delivery of chloroplast content to the vacuole for degradation. These pathways include microautophagy [125,126], senescence-associated vacuoles (SAVs) [127], chloroplast vesiculation (CV)-containing vesicles (CCVs) [128], and MVBs [129] (Figure 4).

#### 6.2.1. Microautophagy

Microautophagy is a type of autophagy characterized by isolation and delivery of cellular components in the lysosome/vacuole for degradation through direct enclosing with the lysosomal/vacuolar membrane [130,131]. There have been a number of reports on plant microautophagy and its involvement in anthocyanin accumulation in the vacuole, and the removal of cellular components such as damaged chloroplasts under starvation. When Arabidopsis plants are exposed to high light levels, the chloroplasts in the mesophyll cells are damaged and are translocated to the vacuole through direct invagination by the tonoplasts [125,126]. During this microautophagy process, a GFP-ATG8-labeled membrane structure is located at the swollen chloroplasts, but unlike autophagosome-mediated pathways, the ATG8-containing membrane structure does not expand to enclose the chloroplasts in microautophagy. In the autophagy-deficient *atg5* and *atg7* mutants, damaged chloroplasts are not engulfed by the vacuole, indicating that the core ATG proteins are required for microautophagy of damaged proteins, even though there is no formation of completed autophagosomes [125,126] (Figure 4). The formation of the ATG8-containing membrane structure at damaged chloroplasts may be involved in the recognition of damaged chloroplasts and/or promoting fusion with the vacuole. 

#### 6.2.2. SAVs

SAVs are induced in chloroplast-containing mesophyll and guard cells during natural or induced leaf senescence [127] (Figure 4). Unlike autophagosomes, SAVs are called vacuoles because they contain a single membrane, are very acidic, and can themselves degrade some of chloroplast components. SAVs contain proteases including SAG12 (SENESCENCE ASSOCIATED 12) and display strong protease activity. During senescence, stromal proteins including Rubisco are transferred to SAVs for degradation. In the autophagy-deficient *atg7* mutant plants, SAVs are still induced under senescence, indicating that their formation is autophagy-independent [127]. The mechanism for senescence-induced biogenesis of SAVs is currently still unknown. 

#### 6.2.3. CCVs

CV is a plastid-targeted transmembrane protein that interacts with a number of chloroplast proteins, probably for destabilizing PSII in thylakoid membranes to facilitate the access of D1 protein by plastidial proteases [128]. CV-containing CCVs are induced under abiotic stress but downregulated by cytokinin. CCVs contain stroma, envelope, and thylakoid proteins but not SAG12, and are not associated with ATG8 [128] (Figure 4). Furthermore, CCV formation is normal in the autophagy-deficient *atg5* mutant [128]. Therefore, CCVs are another type of vesicle-mediated but autophagy-independent pathway for selective degradation of chloroplast molecules in the vacuole. Given that CV is thylakoid membrane protein, CCVs may arise from within the chloroplast.

#### 6.2.4. MVBs

As discussed earlier, MVBs are specialized endosomes in the endocytic pathway whose content is also degraded in the lysosomes/vacuoles. A very recent study uncovers the role of MVBs in the degradation of a chloroplast-destined protein under high temperature to enhance thermotolerance in rice. Through genetic analysis of a thermotolerant African rice variety and a thermosensitive Asian rice variety, Zhang and colleagues identified a quantitative trait locus, *Thermo-tolerance 3* (*TT3*), which consists of two genes, *TT3.1* and *TT3.2* [129]. *TT3.1* encodes a RING-type ubiquitin E3 ligase that is normally localized in the plasma membrane. *TT3.2* encodes a thylakoid-localized chloroplast protein and a negative regulator of thermotolerance whose accumulation leads to the destruction of chloroplasts under high temperature [129]. Under heat stress, plasma membrane-localized TT3.1 ubiquitin E3 ligase is translocated to MVBs, where it ubiquitinates the TT3.2 chloroplast precursor protein and delivers it to the vacuole for degradation [129] (Figure 4). Reduced accumulation of TT3.2 results in the enhanced protection of thylakoids from heat stress and increased heat tolerance. These findings indicate the critical role of MVB-mediated trafficking of chloroplast proteins in plant stress responses. How TT3.2 is sorted into MVBs, instead of being targeted by the UPS, upon ubiquitination by TT3.1 under heat stress is currently unclear.

## 7. Summary and Prospects

Over the past several decades, important progress has been made in the investigation of chloroplast protein quality control and turnover. While the roles of intraplastidic proteases have been long recognized, new information about their dynamic structures, functions, and regulation has expanded steadily over the past two decades. More importantly, a number of extraplastidic pathways have been found to play important roles in chloroplast protein quality control and turnover during normal growth and development, and under diverse stress conditions. These pathways include the UPS-mediated degradation of unimported chloroplast precursors proteins in the cytosol, and chloroplast-associated degradation of chloroplast outer membrane translocon proteins for the regulation of chloroplast protein import. Chloroplast protein quality control and turnover also rely on various types of vesicles including autophagosomes, MVBs, and other structures for trafficking of chloroplast components or even entire chloroplasts to the vacuole for degradation during senescence and stress conditions. As with the ER and mitochondria, UPR in response to accumulated unfolded and misfolded proteins inside chloroplasts has now been firmly established in plants.

Despite these critical developments, there are still many important questions about chloroplast protein quality control and turnover. First, even though a substantial number of mechanisms and pathways have been discovered in chloroplast protein quality control and turnover, they are by no means exhausted, and many critical components in the pathways remain to be identified. For example, turnover of unimported chloroplast precursor proteins in the cytosol is critical not only for the reduction of potentially toxic protein aggregates but also for the regulation of accumulation of specific chloroplast proteins such as rice TT3.2, with an important role in protection of chloroplast structures and functions. However, only a few pathways and factors have so far been identified and functionally analyzed for their roles in targeting chloroplast precursor proteins in the cytosol. The factors involved in the biogenesis of SAVs and CCVs for the delivery of chloroplast components to the vacuole for degradation are currently unknown. Secondly, the diverse pathways and mechanisms in chloroplast protein quality control and turnover likely coordinate, but the underlying mechanisms are unclear. In Arabidopsis, CHIP E3 ubiquitin ligase interacts with and affects the accumulation of specific subunits of plastidic Clp and FtsH protease subunits, but their effects on the structures and activities of plastidic proteases is less clear. Likewise, multiple pathways are involved in the degradation of unimported chloroplast precursors, and diverse vesicle-trafficking pathways participate in the delivery of chloroplast components to the vacuole for degradation. Little information, however, is currently available about the coordination among these pathways for achieving chloroplast proteome homeostasis. Third, the majority of chloroplast protein quality control pathways are induced during specific developmental stages or in response to specific environmental and stress cues. The signaling mechanisms responsible for the activation of these mechanisms and pathways are poorly understood. For instance, critical components responsible for the chloroplast–nuclear communication for the activation of cpUPR, in response to the accumulation of unfolded and misfolded proteins inside the chloroplasts, are largely unknown in higher plants. Finally, most of the published research on chloroplast protein quality control and turnover has been carried out in Arabidopsis; therefore, it is important to expand research into other plants, including important crop plants, to develop important knowledge for the improvement of plant traits.

## Figures and Tables

**Table 1:** Major chloroplast FtsH, Clp and Deg proteases in Arabidopsis.

Protease	Subunit	Type or Unit	Functional Characteristics
FtsH	FtsH1	Type A	Thylakoid-localized ATP-dependent metalloprotease complex; participate in PSII repair, including D1 turnover
	FtsH5		
	FtsH2	Type B	
	FtsH8		
Clp	ClpC1	Chaperone unit	Stromal ATP-dependent serine protease complex; targets multiple chloroplast proteins including transporters and metabolic enzymes; participate in the quality control of imported proteins across the inner envelope.
	ClpC2		
	ClpD		
	ClpP3	P ring	
	ClpP4		
	ClpP5		
	ClpP6		
	ClpS	Adaptor	
	ClpF		
	ClpP1	R ring	
	ClpR1		
	ClpR2		
	ClpR3		
	ClpR4		
	ClpT	Accessary	
	ClpT2		
Deg	Deg1	Thylakoid lumen	Participate in PSII repair including degradation and D1 and light-harvesting proteins.
	Deg5		
	Deg8		
	Deg2	Stromal side of thylakoid	
	Deg7	Stroma	

## Data Availability

Not applicable.
